# Determination of medical abortion eligibility by women and community health volunteers in Nepal: A toolkit evaluation

**DOI:** 10.1371/journal.pone.0178248

**Published:** 2017-09-07

**Authors:** Kathryn Andersen, Mary Fjerstad, Indira Basnett, Shailes Neupane, Valerie Acre, Sharad Kumar Sharma, Emily Jackson

**Affiliations:** 1 Ipas, Chapel Hill, NC, United States of America; 2 WomanCare Global, 8910 University Center Lane, San Diego, CA, United States of America; 3 Independent Consultant, Kathmandu, Nepal; 4 Valley Research Group (VaRG), Lalitpur, Nepal; 5 Independent Consultant, Los Angeles, CA, United States of America; University of Cape Town School of Public Health and Family Medicine, SOUTH AFRICA

## Abstract

**Objective:**

To determine if pregnant, literate women and female community health volunteers (FCHVs) in Nepal can accurately determine a woman’s eligibility for medical abortion (MA) using a toolkit, compared to comprehensive abortion care (CAC) trained providers.

**Study design:**

We conducted a prospective diagnostic accuracy study in which women presenting for first trimester abortion, and FCHVs, independently assessed each woman’s eligibility for MA using a modified gestational dating wheel to determine gestational age and a nine-point checklist of MA contraindications or cautions. Ability to determine MA eligibility was compared to experienced CAC-providers using Nepali standard of care.

**Results:**

Both women (n = 3131) and FCHVs (n = 165) accurately interpreted the wheel 96% of the time, and the eligibility checklist 72% and 95% of the time, respectively. Of the 649 women who reported potential contraindications or cautions on the checklist, 88% misidentified as eligible. Positive predictive value (PPV) of women’s assessment of eligibility based on gestational age was 93% (95% CI 92, 94) compared to CAC-providers’ (n = 47); PPV of the medical contraindications checklist and overall (90% [95% CI 88, 91] and 93% [95% CI 92, 94] respectively) must be interpreted with caution given women’s difficulty using the checklist. PPV of FCHVs’ determinations were 93% (95% CI 92, 94), 90% (95% CI 89,91), and 93% (95% CI 91, 94) respectively.

**Conclusion:**

Although a promising strategy to assist women and FCHVs to assess MA eligibility, further refinement of the eligibility tools, particularly the checklist, is needed before their widespread use.

## Introduction

Nepal’s high abortion-related maternal mortality rate (MMR) of 770 deaths per 100,000 live births in 1990 [1] was key in liberalizing the country’s restrictive abortion law in 2002. After legal reform, Nepal rapidly introduced and scaled-up safe abortion services. By 2011, 532 sites and more than 1,500 providers had been Comprehensive Abortion Care (CAC)-trained and certified [2]. Despite a more than 4-fold decline in MMR by 2010 [1], abortion remains Nepal’s third highest cause of maternal mortality, accounting for 14% of direct maternal hospital deaths [3]. Providing abortion services to Nepal’s 28 million people is uniquely challenging-in addition to poverty and prolonged political instability, rugged mountainous terrain limits health services access, particularly in geographically isolated areas. Services are largely confined to urban and district-level facilities, leaving Nepal’s large rural population, where the greatest reductions in MMR are needed, with a several day walk to services [4]. More than 21 million unsafe abortions occur globally every year, 98% of them in resource poor countries [5]. While numerous barriers limit access to safe abortion, lack of trained providers is one of the most critical, disproportionately affecting women who live in rural areas, are poor, less educated, young and unmarried [5]. A 2010 study of Nepali women receiving treatment for abortion complications after legalization found that 81% of medical abortions were obtained from uncertified sources, and 89% of women had ingested unsafe, ineffective or unknown substances to induce their abortion [6].

Medical abortion (MA) is highly effective with low complication rates up to, and after, 63 days gestation [7–12]. Particularly in low-resource settings with limited health care access and few trained providers, shifting MA provision to additional cadres of providers, or potentially to women themselves, can increase safe abortion access, and decrease unsafe abortion morbidity and mortality. Multiple studies have established the safety and efficacy of non-physician providers of medical abortion [13, 14], and the World Health Organization (WHO) recommends that, in addition to specialist and non-specialist doctors, associate clinicians, midwives, nurses, auxiliary nurses and auxiliary nurse midwives can provide MA when properly trained [15]. WHO makes no recommendation regarding provision of MA by pharmacists or lay health workers, instead calling for research documenting the safety, effectiveness and feasibility of approaches to expand the role of these two groups in performing components of MA. Indeed, only one published study, conducted in Ethiopia, South Africa and India, has examined community health workers’ ability to determine women’s eligibility for MA [16]. Using a toolkit consisting of a pregnancy test, a gestational age wheel and a checklist of potential contraindications to MA, these workers accurately determined women’s MA eligibility between 92–77% of the time. No studies have evaluated women’s ability to self-assess for MA eligibility. However, where abortion is restricted or unavailable, women already access MA drugs, using them without eligibility assessment or proper instruction in their use [17, 18]. Despite lack of assessment by a trained provider, abortion procured in this way appears to pose less risk to women than unsafe use of instruments or traditional, non-effective treatments [5, 17].

Based on strong clinical evidence, recognition of the need to improve access, and political support to do so, provision of MA up to 63 days gestation has already expanded in Nepal from doctors to nurses and auxiliary nurse midwives trained as birth attendants [19, 20][19, 20][19, 20]. To explore the feasibility and safety of further expanding MA services to the community level, we evaluated the ability of literate women, and a cadre of minimally trained female community health volunteers (FCHVs) in Nepal to independently assess women’s eligibility for MA, based on: (1) determination of pregnancy duration using a gestational dating wheel, and (2) screening for MA drug contraindications using a checklist. We compared women’s and FCHVs’ assessments of eligibility, respectively, to assessments made by trained, registered CAC-providers using Nepal’s current standard of care.

## Methods

### MA eligibility tools

**Fig 1:**
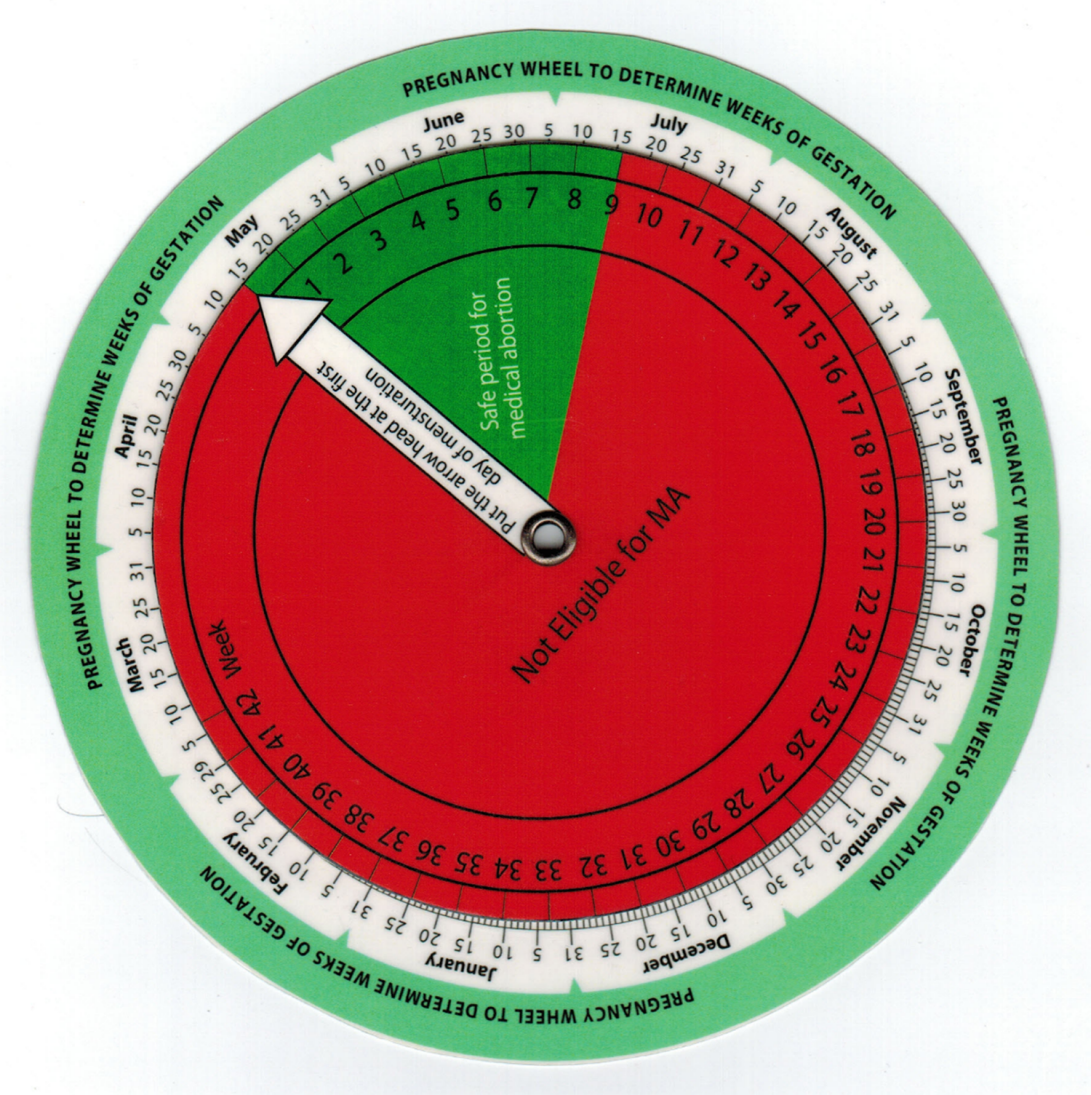
Modified gestational dating wheel. To use the wheel, turn the wheel until the arrow points at the first day of the woman’s reported LMP, then find the current date on the wheel. If the date is in the green zone (here, from May 15 to July 15) the woman is eligible for MA based on the gestational age of the pregnancy (< 63 days). If the date is in the red zone, she is not eligible. (Note: the version used in the study included the Nepali calendar and all instructions written in in Nepali).

**Fig 2:**
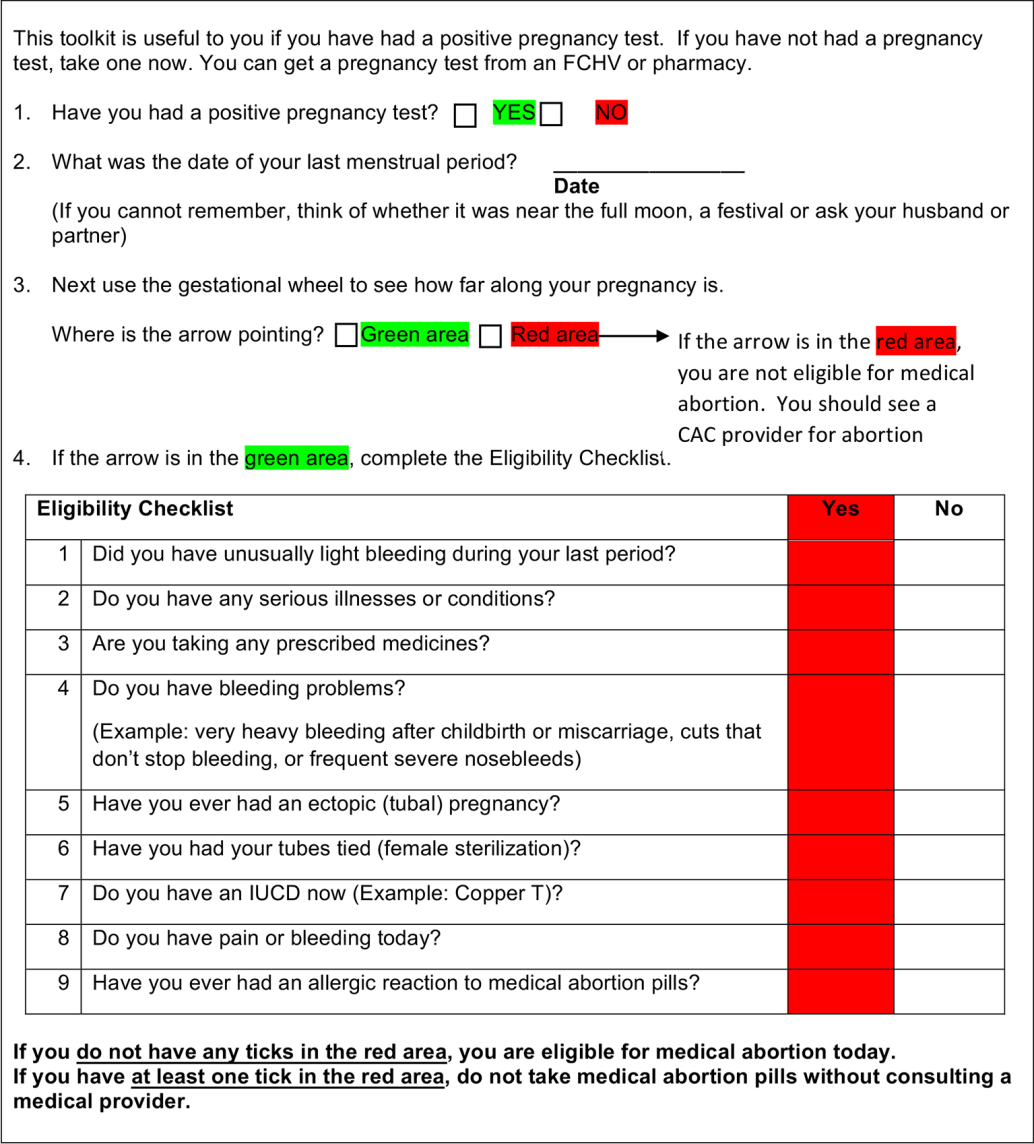
MA eligibility checklist. The user answers the questions in order. The first portion of this worksheet is intended to be used with the gestational dating wheel (see Fig 1). The second portion is the eligibility checklist. A response in a red area for any question indicates that the woman requires further evaluation prior to MA.

The eligibility tools consist of a modified gestational dating wheel and a checklist (Figs 1 and 2). The tools are aimed at identifying those women who could use MA without evaluation from a CAC-provider (eligible), and those who require CAC-provider evaluation before MA (ineligible). The wheel estimates pregnancy duration based on women’s reported last menstrual period (LMP). Pregnancies less than 63 days fall in a green shaded area, indicating the pregnancy is sufficiently early for MA (eligible); these women are instructed to complete the checklist. The rest of the wheel is shaded red (ineligible). The checklist asks nine health related questions assessing contraindications (eg. drug allergy or ectopic pregnancy) or medical issues that could potentially be problematic (eg. risk for anemia, called ‘cautions’ in this paper) when using MA drugs, and that may benefit from CAC-provider evaluation prior to MA. Any ‘yes’ answer on the checklist results in a tick in a red-shaded box; any red ticks prompt consultation with a CAC-provider (ineligible). In addition to using the wheel and completing the checklist if indicated, women and FCHVs were also asked to make their own, overall eligibility assessment by answering the question: “Do you think you are/she is eligible for medical abortion today?”

All tools were translated into Nepali, and the gestational dating wheel was further adapted to reflect the Nepali calendar. Translated tools were tested for construct validity, and underwent three rounds of pretesting by Nepali women, FCHVs and CAC-providers prior to study use. Tools were edited following each round of pretesting based on results and feedback; pretesting was considered complete when no further substantive comments were elicited.

### Study participants

We recruited women ≥ 16 years old, with a positive pregnancy test, who sought safe abortion at one of seven study facilities, and were able to read and write in Nepali. Literacy was assessed by women’s self-report and demonstrated ability to read and understand an item from the checklist.

Nepal boasts a strong network of more than 48,000 FCHVs. FCHVs receive 18 days of training in maternal and child health, and those in select districts receive additional training in early pregnancy diagnosis with urine pregnancy tests, and referral to antenatal care, safe abortion or family planning as appropriate [21]. They are a trusted source of maternal and child health information, and a key referral link between their communities and health services. FCHVs are not compensated for their work, and work five hours/week on average. We randomly selected literate FCHVs previously trained in the early pregnancy diagnosis and referral program from the same six districts where this study was carried out. One FCHV was assigned to each of the seven study facilities for one to two weeks during data collection.

CAC-providers included doctors and staff nurses trained and registered with the Nepal Ministry of Health to provide CAC with manual vacuum aspiration and MA. We offered participation to all CAC-providers at the included study facilities.

### Study facilities

We selected seven study facilities in six Nepali districts based on abortion caseload. All were located in urban centers, and drew patients from surrounding rural areas. In the Central region, study facilities included one public hospital and one non-governmental (NGO) clinic in Kathmandu, one public hospital in Chitwan District, and one NGO clinic in Lalitpur District. One public hospital and one NGO clinic were included from the Rupendehi and Kaski districts (Western region), and one NGO clinic was included from the Jhapa District (Eastern region).

### Study procedures

A locally based, trained study coordinator was assigned to each study facility for the duration of the study. Coordinators were drawn from a pool of experienced local data collectors, and underwent a four-day orientation to the study, including study design, data collection procedures and assurance of ethical standards. Study coordinators were responsible for orienting each FCHV to the eligibility tools at the beginning of her assigned one- or two-week posting. This orientation lasted approximately one hour, included an explanation of the study, a review of the tools, and one or more case studies for practice. This orientation was intended to mimic training that FCHVs would receive were such a toolkit put into practice. Coordinators collected FCHV demographic data at the beginning of their postings, and the FCHV’s impressions of the tools at the conclusion.

Study coordinators additionally oriented each woman to the toolkit, as well as obtained informed consent to participate in the study. This verbal orientation lasted approximately 15 minutes, during which time the coordinator confirmed the woman’s literacy by having her read a checklist item, and explained how to use the gestational dating wheel. Each woman then self-assessed her eligibility for MA using the tools, and answered a questionnaire regarding her impression of the toolkit’s usability, and demographic data. Women’s responses were sealed in an envelope, and women were asked to not share their self-assessment with the FCHV or CAC-provider they would subsequently meet. Next, using the tools, an FCHV independently assessed the woman’s eligibility for MA. The completed FCHV assessment was sealed in a second envelope. Finally, a CAC-provider independently assessed the woman’s eligibility using standard of care, and completed a brief questionnaire about her eligibility. In Nepal, the standard of care to establish MA eligibility includes history, physical examination (including pelvic examination) and laboratory tests as needed. Ultrasound is available in only a small number of Nepal’s abortion care facilities.

### Data management and analysis

Data from data collection forms were translated into English and de-identified data were entered into Visual Foxpro 2.6, and checked for consistency and completeness. Data were analyzed with SPSS version 2.0 and Stata 11.1.

Comprehension of the eligibility tools was assessed by comparing the wheel and checklist information recorded by women and FCHVs to their interpretation of that information. In other words, if a woman or FCHV entered a tick mark into a red box, did that women or FCHV correctly interpret that tick mark to mean she was ineligible.

Assessment of MA eligibility based on gestational age determination using the wheel, the checklist, and the overall assessment of eligibility was compared between women and CAC-providers, and FCHVs and CAC-providers. We analyzed agreement between women and CAC-providers and FCHVs and CAC-providers by using 2x2 tables and diagnostic test statistics (positive predictive value [PPV], negative predictive value [NPV], sensitivity [Sn] and specificity [Sp]), where women/FCHVs correctly identifying eligibility was considered a positive test.

### Ethical considerations

This study was reviewed by the Allendale Institutional Review Board in the United States and the Nepal Health Research Council in Nepal. All participants provided written, informed consent to participate. As the legal age of majority and consent in Nepal is 16, women ≥ 16 were eligible for the study.

## Results

We enrolled 3131 women (Table 1) between September 2013 and February 2014; an additional 1212 women were excluded (four women were not pregnant; all others were illiterate). Enrolled women were younger, more educated, from more advantaged social groups and had fewer pregnancies than excluded women (data not shown, p<0.001). Study FCHVs (n = 165) had worked about 13 years, and overall were less educated than enrolled women.

**Table 1:** Participant sociodemographic data.

	Women	FCHVs
	n = 3131	n = 165
Age, mean (SD)	27.2 (5.4)	38.2 (6.9)
Pregnancies, mean (SD)	2.7 (1.3)	N/A
Some secondary school or higher, n (%)	2814 (90)	73 (44)
Married, n (%)	2981 (95)	N/A
Caste/Ethnicity, n (%)		
Disadvantaged Groups*	1013 (32)	24 (15)
Relatively Advantaged	530 (17)	22 (13)
Upper Caste Groups	1588 (51)	119 (72)
Literacy, n (%)	3131 (100)	165 (100%)
Experience as FCHV, mean years (SD)	N/A	12.6 (7.3)

SD = standard deviation

*Disadvantaged groups includes dalit, disadvantaged janajaties, disadvantaged non-dalit Terai caste groups, and religious minorities.

### Comprehension and ease of use of MA eligibility tools by women and FCHVs

Complete wheel and LMP data were available for 3094 women. We excluded women who did not record their LMP (n = 15), the color of their wheel assessment (n = 16), or both (n = 6) from this analysis. We found 96% agreement between women’s recorded LMP and their eligibility self-determination using the wheel. Ninety-five women with gestations ≤63 days by LMP incorrectly identified as ineligible using the wheel (3% of n = 3094), and 26 women (1% of n = 3094) with gestations >63 days by LMP incorrectly identified as eligible.

Among women who completed the checklist (n = 2723), we found 71% agreement between women’s interpretation of their responses and their eligibility as determined by the checklist. Of the 2074 women with no red items marked, 216 (8% of n = 2723) incorrectly identified as ineligible when interpreting their results; of the 649 women who marked red items, 568 (21% of n = 2723) incorrectly identified as eligible.

FCHVs correctly interpreted the wheel 96% of the time. They more accurately interpreted the checklist than did women (95% agreement); only 93 FCHV-collected checklists with no red items (3% of n = 2740) were incorrectly identified as ineligible, and 59 of the 527 FCHV-collected checklists with red items marked (2% of n = 2740) were incorrectly identified as eligible by FCHVs.

Despite their demonstrated difficulty interpreting the eligibility tools, 91% of women and 97% of FCHVs stated the wheel and checklist were easy to use. No patterns in misinterpretation were observed based on the questions or women’s characteristics (data not shown).

### MA eligibility assessment among women and CAC-providers

Agreement between women and CAC-providers was 83% for overall eligibility (based on the answer to the question “Do you think you are eligible for medical abortion today?”); agreement was higher for eligibility based on gestational age assessment using the wheel (87%) and lower for eligibility based on medical contraindications determined using the checklist (71%) (Table 2). PPV for women’s assessment of overall eligibility and eligibility based on the wheel were both 93% (95% CI: 92, 94), while PPV for the checklist was lower (90%, 95% CI: 88, 91). For all eligibility assessments, NPV of women using the toolkit was poor.

**Table 2:** Women’s self-assessments of their overall eligibility, eligibility based on gestational age determination using the dating wheel, and medical eligibility using the checklist for MA drug use compared to CAC-provider’s determinations of their eligibility based on standard of care.

	Provider Assessment Eligible		Provider Assessment Ineligible		Total		PPV	NPV	Sn	Sp
	n	%^a^	n	%^a^	n	%^a^	% (95%, CI)	% (95% CI)	% (95% CI)	% (95% CI)
**Gestational Age Assessment**							93 (92, 94)	56 (52, 60)	91 (90, 92)	62 (58, 66)
Women Assessment-Eligible	2378	77	182	6	2560	83				
Women Assessment-Ineligible	234	8	297	10	531	17				
Total	2612	85	479	16	3091^b^	100				
**Medical Contraindications Assessment**							90 (88, 91)	12 (9, 15)	76 (75, 78)	26 (21, 32)
Women Assessment-Eligible	1852	68	214	8	2066	76				
Women Assessment-Ineligible	572	21	76	3	648	24				
Total	2424	89	290	11	2714^c^	100				
**Overall Eligibility Assessment**							93 (92, 94)	46 (43, 50)	87 (85, 88)	64 (59, 68)
Women Assessment-Eligible	2277	73	175	6	2452	79				
Women Assessment-Ineligible	354	11	307	10	661	21				
Total	2631	85	482	16	3113^d^	100				

PPV = positive predictive value, NPV = negative predictive value, Sn = sensitivity, Sp = specificity

^a^Numbers may not add up to 100% due to rounding.

^b^40 women excluded: 18 women missing provider assessment of gestational age and 22 women missing gestational wheel assessment.

^c^417 women excluded: 9 women missing provider assessment, 408 women with missing or inconclusive checklists. Of note, women who determined themselves ineligible after using the dating wheel were instructed not to complete the checklist.

^d^18 women excluded: missing provider assessment.

**Fig 3:**
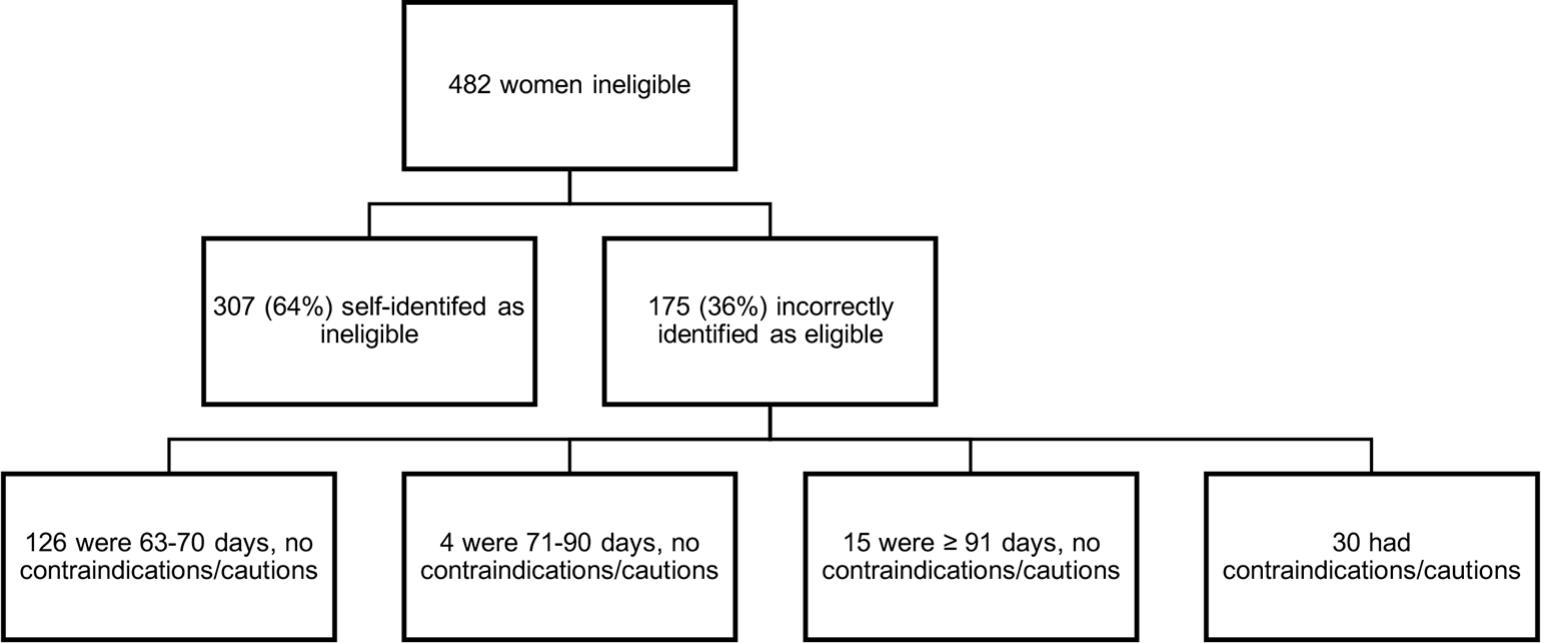
Clinical risk for the 482 women (15% of n = 3113) who were determined to be ineligible for medical abortion by comprehensive abortion care providers.

In their assessment of overall eligibility, 175 women (6%) believed themselves eligible when the CAC-provider determined they were ineligible–these women were potentially at increased clinical risk if using MA unsupervised. The majority (n = 126, 4% of n = 3113) were 63–70 days gestation with no cautions for MA drug use (Fig 3). The remaining 49 women (2% of n = 3113) had potential for increased risk if using MA unsupervised-19 were >77 days gestation, and 30 (1% of n = 3113) had cautions for MA drug use as determined by CAC-providers.

While only one woman had a true contraindication to MA drug use (allergy), 41 women had cautions for MA drug use, either by their own or CAC-provider assessment (Table 3). In our study, CAC-providers determined contraindications and cautions based on their understanding of standard of care and good practice. In fact, most of the cautions reported by CAC-providers are not conditions that would prevent women from safely using MA.

**Table 3:** Contraindications and cautions (conditions that require further evaluation by a CAC-provider before MA drug use) reported by women or determined by CAC-provider (n = 42^a^).

**Contraindications**	**n**	**%^b^**
Medication allergy	1	2
**Cautions**	**n**	**%**
Bleeding risk (as noted by provider)	5	12
Diabetes	6	14
Pelvic inflammatory disease	5	12
Molar cyst	4	10
Anemia	2	5
Current hepatitis B medication use	2	5
Kidney problem	2	5
Rheumatic heart disease	1	2
Jaundice	1	2
Diarrhea	1	2
Not specified	12	29

^a^Includes 30 women who believed they were eligible and CAC-provider deemed them ineligible, and 12 women for whom both woman and CAC-provider agreed she was ineligible.

^b^Numbers may not add up to 100% due to rounding.

### MA eligibility assessment among FCHVs and CAC-providers

FCHV results using the Eligibility Tools were similar to women’s self-assessment. Agreement for MA eligibility between FCHVs and CAC-providers was 75% (Table 4), and 5% of women determined eligible by FCHVs were deemed ineligible by CAC-providers.

**Table 4:** FCHV’s assessments of a woman’s overall eligibility, eligibility based on gestational age determination using the dating wheel, and medical eligibility using the checklist for MA drug use compared to CAC-provider’s determinations of their eligibility based on standard of care.

	Provider Assessment Eligible		Provider Assessment Ineligible		Total		PPV	NPV	Sn	Sp
	n	%^a^	n	%^a^	n	%^a^	% (95%, CI)	% (95% CI)	% (95% CI)	% (95% CI)
**Gestational Age Assessment**							93 (92, 94)	58 (53, 62)	92 (91, 93)	59 (55, 64)
FCHV Assessment-Eligible	2405	78	192	6	2597	84				
FCHV Assessment-Ineligible	205	7	279	9	484	16				
Total	2610	85	471	15	3081^b^	100				
**Medical Contraindications Assessment**							90 (89, 91)	12 (10, 16)	81 (80, 83)	22 (18, 28)
FCHV Assessment-Eligible	1990	73	226	8	2216	81				
FCHV Assessment-Ineligible	461	17	65	2	526	19				
Total	2451	89	291	11	2742^c^	100				
**Overall Eligibility Assessment**							93 (91, 94)	34 (31, 37)	77 (75, 79)	66 (61, 70)
FCHV Assessment-Eligible	2021	65	162	5	2183	70				
FCHV Assessment-Ineligible	601	19	313	10	914	30				
Total	2622	85	475	15	3097^d^	100				

PPV = positive predictive value, NPV = negative predictive value, Sn = sensitivity, Sp = specificity

^a^Numbers may not add up to 100% due to rounding.

^b^50 women excluded: 18 women missing provider assessment of gestational age and 32 women missing FCHV gestational wheel assessment.

^c^389 women excluded: 8 women missing provider assessment, 381 women with missing or inconclusive checklists. Of note, women who determined themselves ineligible after using the dating wheel were instructed not to complete the checklist.

^d^34 women excluded: 18 women missing provider assessment and 16 women missing FCHV assessment.

## Discussion

Our study is the first to examine women’s ability to self-determine MA eligibility using a toolkit consisting of a gestational dating wheel and a checklist. We found that 96% of women were able to use and interpret the gestational dating wheel correctly; only 4% of women misinterpreted the results of the wheel. Compared to CAC-providers using standard of care to determine eligibility, we found a PPV of 93% for women using the wheel; 6% of women believed they were eligible based on gestational dating when CAC-providers determined they were not.

However, women were less successful using the eligibility checklist. Nearly a third of women (29%) did not correctly interpret their own responses. Most concerning, of the 649 women who marked red items, indicating that they should identify as ineligible due to potential medical contraindications to MA drugs, 88% incorrectly identified as eligible. The observed PPV of the checklist and the overall eligibility question was high-90% and 93%, respectively-despite this, due to the fact that very few women are actually contraindicated to use MA drugs. Indeed, less than 1% of women in our study had true MA drug contraindications. The observed high PPV (93%) of the overall eligibility assessment question, “Do you think you are eligible for medical abortion today?”, is also likely due to the fact that few women are truly ineligible for MA.

Women’s demonstrated difficulty using the checklist was despite three rounds of pretesting during which it was well understood, and the fact that 92% of women in our study reported that the checklist was easy to use. We found no discernible patterns in misinterpretation based on the questions or women’s characteristics. Most likely is that women did not understand how to interpret a tick in a red box, a problem which could be improved with future versions. Similarly, the addition of an ‘I don’t know’ option or a ‘no/I don’t know’ option may help women to more accurately and easily complete the checklist, particularly if comprehension of questions was an issue [22, 23].

FCHVs interpreted the eligibility checklist more accurately than did women; we found 95% agreement between information entered into FCHV-collected eligibility checklists and the interpretation by the FCHVs. Like women, FCHVs used the gestational dating wheel well. Compared to CAC-providers, we found a PPV of 93%, 90% and 93% for the wheel, checklist, and overall eligibility question when used by FCHVs. FCHVs and CAC-providers agreed regarding MA eligibility 75% of the time; these data are similar to a 2016 study conducted in Ethiopia, India and South Africa that found that 711 community health workers could use a similar toolkit (dating wheel and checklist) to accurately determine women’s MA eligibility between 92–77% of the time [16]. That study found that health workers with higher levels of training and experience with diagnostic aids used the toolkit more accurately; it is likely that practice with the tools during multiple client encounters helped our FCHVs gain experience with the tools and use them more accurately. These data are promising for the use of these or similar tools in a facilitated fashion; that is, an individual trained in the use of the tools, such as an FCHV, community health worker, pharmacist, or youth advocate, could work with a woman to assess her eligibility. Particularly in remote areas with few trained health providers, expansion of MA in this fashion could increase safe abortion access. However further refinement of such tools, and determination of optimal training for their accurate use, is needed.

It is important to note that CAC-providers in our study were more conservative than current guidelines when assessing contraindications to MA drugs, resulting in more women identified as medically ineligible than necessary. Only one of the 42 women deemed ineligible by CAC-providers based on medical conditions had a true MA drug contraindication-allergy. Had CAC-providers more closely conformed to current guidelines regarding MA contraindications, 16 of the 175 women who self-assessed as eligible, but CAC-providers deemed ineligible, would have been eligible for MA. Our CAC-providers would benefit from retraining in MA contraindications to ensure that all eligible women requesting MA drugs receive them. Prior to future studies, retraining of providers in the latest CAC guidelines prior to study inception would be beneficial.

Our study has several important methodologic limitations, in addition to those outlined above. Most notably, our tools require users be literate to use them. Women in our study were uniformly literate, and had more education than excluded women. Illiterate women are unable to use the tools in their current form without assistance, and literate women with less education than our sample, or who receive less instruction in tool use, may have more difficulty than we observed. Given the concentration of unsafe abortion related morbidity and mortality among rural women with less education, development and testing of lower literacy or pictorial tools could potentially more effectively reach those women most at risk. To compare assessments made by women and FCHVs to CAC-providers, we conducted our study in urban, clinical settings where those providers work. Although these facilities draw patients from the surrounding rural areas, this limits the generalizability of our findings to rural and more remote areas, and to other types of health care settings. Finally, small numbers of women ineligible for MA, 11% in our study based on CAC-provider determination of medical contraindications, limits the NPV of these tools.

A key question to consider in task-shifting MA to community or lay health workers, or to women themselves, is what level of error in assessment of eligibility is acceptable? Particularly in places where access to abortion is limited, increasing MA availability could drastically reduce abortion related morbidity and mortality, even when a small proportion of women are incorrectly assessed as eligible. Indeed, this has been observed in countries where women already use MA drugs-often clandestinely and without adequate information-without the assistance of health care providers [24, 25]. Given the rarity of true medical contraindications, more concerning may be outpatient use of MA drugs by women beyond 70 days gestation. In this scenario, there are two potential outcomes: due to decreasing efficacy of MA drugs in the late first trimester, and different recommended regimens of MA drugs in the second, such use will either not induce an abortion, or women could have a later abortion at home [15]. In their recent commentary, Raymond et al. [26] argue that serious harm from use of MA drugs in the late first trimester is rare, citing a study [12] in which 90% of women between 70–83 days gestation treated with MA drugs successfully aborted, and only one serious complication (bleeding requiring transfusion) was observed. They further argue that in some cases, risk of a late, at-home abortion may be considered acceptable, as in pretreatment of women having second trimester abortion procedures with digoxin, despite the possibility of extramural expulsion [27]. Regardless, numbers of women at risk is still small; in our study, less than 1% of women identified themselves as eligible for MA with a gestation >70 days. A recent analysis [26] concluded that using a more conservative cut-off value for to determine MA eligibility based on gestational age, such as <56 days, could result in as few as 0.6% of women being offered MA beyond gestational age limits. Future iterations of our wheel should explore if changing these gestational age eligibility parameters results in fewer ineligible women identifying as eligible based on pregnancy duration. The balance of risks and benefits when considering use of MA drugs with less clinical supervision remains an area of active discussion and research.

In conclusion, women in our study were able to use a dating wheel to determine their MA eligibility based on gestational age, but were not able to effectively use a checklist to determine if they had medical contraindications to MA drugs. FCHVs were able to use both the dating wheel and the checklist well, compared to trained CAC-providers using Nepali standard of care. Despite limitations of our toolkit, providing women and health volunteers with simple guidance to determine eligibility for MA remains a promising approach for the safe use of MA drugs with less supervision. Future research should focus on refining the tools, particularly the checklist, so that women are more successful in identifying their ineligibility. Implementation of such a toolkit more broadly can increase women’s access to safe abortion at the community level, either by using a revised toolkit alone or with the assistance of a trained volunteer or other health worker. Given limited access to safe abortion in rural settings, in Nepal and other countries, such an expansion of safer services could have profound effects in those areas where high maternal mortality due to unsafe abortion persists.
